# Occupational sedentary behaviour and mental health symptoms among software and IT workers in China: a cross-sectional study using path analysis

**DOI:** 10.1186/s12889-026-26761-2

**Published:** 2026-04-08

**Authors:** Ming Jin, Michelle Swainson, Abigail Morris

**Affiliations:** 1 Lancaster University, Lancaster, UK; 2 Faculty of Health and Medicine, Lancaster University, Health Innovation One, Sir John Fisher Drive, Lancaster, LA1 4AT UK

**Keywords:** Occupational sedentary behaviour, Mental health, Software and IT industry, Office workers, Path analysis

## Abstract

Evidence regarding the association between occupational sedentary behaviour and common mental health symptoms remains limited. As the workplace contributes substantially to daily sedentary time, understanding its association with employees’ mental health is important. This study aimed to: (1) estimate self-reported sedentary behaviour among software and information technology (IT) workers in China; (2) examine the association between occupational sedentary behaviour and common mental health symptoms; and (3) identify factors that may influence this relationship.

A cross-sectional online survey was conducted among software and IT employees from four companies in Wuhan, China. Self-reported data on occupational sedentary time and mental health symptoms were collected. Hierarchical regression and path analysis were used to explore associations among 235 valid responses.

Mean occupational sedentary time was 427.9 (± 133.2) minutes (≈ 7.13 ± 2.22 hours), while total daily sedentary time reached 499.9 (± 161) minutes (≈ 8.33 ± 2.68 hours) during workdays. Participants reported moderate to high levels of depression and anxiety, and moderate to low levels of stress. After adjustment for confounding variables, no significant associations were observed between total or occupational sedentary behaviour and depression or anxiety. Total sedentary time was significantly associated with stress. The association between occupational sedentary behaviour and stress was attenuated after accounting for occupational factors and poor sleep quality. Path analysis, based on a cross-sectional model in which sleep quality was specified a priori and occupational factors were explored in a data-driven manner, suggested a potential indirect association between occupational sedentary behaviour and stress. Longer tenure appeared to act as a confounding factor, being negatively associated with both occupational sedentary behaviour and stress.

Software and IT workers in China exhibited high levels of occupational sedentary behaviour and pre-clinical mental health symptoms. No direct associations were identified between occupational sedentary behaviour and depression, anxiety, or stress in this cross-sectional sample. However, a potential indirect association with stress via poor sleep quality was observed but was not statistically significant. These findings are exploratory and hypothesis-generating rather than causal, and may help inform future longitudinal research and workplace initiatives focusing on sleep hygiene and holistic well-being.

**Supplementary Information:**

The online version contains supplementary material available at 10.1186/s12889-026-26761-2.

## Introduction

Excessive (≥ 6 hours) daily sedentary behaviour (including leisure and occupational time) is associated with a range of adverse health outcomes, including increased incidence of type 2 diabetes, cardiovascular disease and cancer mortality [1]. From a biological perspective, prolonged sedentary behaviour has been associated with metabolic dysregulation, including altered glucose and lipid metabolism and low-grade inflammatory processes [2, 3]. Moreover, recent studies show that prolonged daily sedentary behaviour (≥ 6 hours) is also deleterious to common mental health conditions, including depression and anxiety [4, 5]. However, current evidence is largely based on leisure-time sedentary behaviour. While recent syntheses have begun to explore the occupational context [6], the association between occupational sedentary behaviour and common mental health symptoms is less understood [7].

Given that the workplace is an important setting where high volumes of daily sedentary behaviour are accumulated [8], it is essential to understand whether there is an association between occupational sedentary behaviour and mental health symptoms. Depending on job role, work typically accounts for 60%−90% of an individual’s daily sitting time [9, 10]. Additionally, poor mental health can detrimentally affect work performance and productivity, resulting in a reduced pace, an increase in errors, and increased absenteeism [11, 12]. These consequences not only affect individual employees’ well-being but also have economic implications for organisations, manifesting in decreased productivity [13].

The Software and IT workforce, characterised by desk-based and computer-led tasks, is prone to a high prevalence of occupational sedentary behaviour [14, 15]. China hosts the world’s largest Software and IT workforce, comprising over 9.4 million professionals in this sector [16]. Notably, China has experienced a noticeable increase in the prevalence of mental health disorders over the past three decades, coinciding with swift economic development [17]. It is estimated that the prevalence of mental health disorders has reached as high as 16.6% [18], suggesting that approximately one in six individuals in the Chinese population may experience mental health disorders during their lifetime.

However, to date, there is currently no data about the occupational time spent sedentary in this population of Software and IT workers in China. Moreover, the association between occupational sedentary behaviour and common mental health symptoms has not been explored among this significant workforce. Therefore, the aims of this study were:

1. To estimate the duration of total and occupational sedentary behaviour and the prevalence and severity of mental health symptoms among Software and IT workers in China.
2. To determine whether there is an association between occupational sedentary behaviour and common mental health symptoms.
3. To identify variables that may influence the association between occupational sedentary behaviour and common mental health symptoms.

## Methods

### Research design and setting

A cross-sectional study targeting employees in the Software and IT industry in Wuhan, China. Ethical approval was obtained from Lancaster University’s Faculty of Health and Medicine Ethics Committee in accordance with the Declaration of Helsinki (reference: FHM-2024-4276-RECR-3).

### Participant and procedure

Data collection was conducted between May-August 2024. Four companies within the Software and IT industry in Wuhan, China, were recruited using convenience sampling through professional networks and direct outreach. Following the acquisition of organisational consent, a nominate gatekeeper in each company distributed the survey invitation and link to employees via email or WeChat, given its prevalence in Chinese workplace communication [19].

Individuals were eligible if they were aged 18 or above, employed full-time in a Software and IT role, and worked in a company-based office setting. Exclusion criteria included part-time or intern employment and primary home-based or remote work arrangements. Participants were not excluded based on the presence of diagnosed mental disorders or other health conditions. Mental health outcomes were assessed using self-reported, non-diagnostic instruments, capturing symptom levels within a currently employed working population.

Participants accessed the survey via Qualtrics and completed a digital consent form before proceeding. Completing the questionnaires took approximately 15 minutes.

### Total and occupational sedentary behaviour on workdays

The short-form International Physical Activity Questionnaire (IPAQ) was used to estimate total sedentary behaviour. It asks: “During the last 7 days, how much time did you spend sitting on a week day?” [20]. The IPAQ has been validated as a reliable and valid tool among the Chinese population [21, 22]. Participants were asked to recall their sitting time on weekdays during the past 7 days, including work, home, coursework, and leisure time, and to report it in hours and minutes. Subsequently, for occupational sedentary behaviour, participants were asked to recall their sitting time specifically for work on weekdays during the past 7 days, and again to report it in hours and minutes. The responses were converted to minutes per day by multiplying reported hours by 60 and adding the reported minutes for data analysis.

### Depression and anxiety

Sensations of depression and anxiety were measured using the Hospital Anxiety and Depression Scale (HADS) [23]. While initially developed to detect anxiety and depression in hospital medical outpatient clinics, this scale has been widely adopted as a screening tool rather than a diagnostic instrument [24, 25]. It has been validated in the general population, and recent studies also support its use in workplace settings [26, 27]. Moreover, it is widely used and validated among the Chinese population [28, 29].

The HADS contains 14 items, with 7 measuring symptoms of anxiety (e.g., “I get sudden feelings of panic”) and 7 measuring symptoms of depression (e.g., “I feel cheerful”), which are scored separately [23]. Each item was assigned a value from 0 to 3, resulting in a total score that ranges from 0 to 21 for each scale. A score of 0–7 represents no symptoms of depression or anxiety, 8–10 could indicate potential signs of anxiety or depression feelings, while a score of 11 or higher may indicate a higher likelihood of experiencing depression or anxiety symptoms [23]. This scale demonstrated acceptable reliability in this study, with Cronbach’s α values of 0.839 for the overall scale, 0.811 for the anxiety subscale, and 0.704 for the depression subscale.

### Stress

Psychological stress was assessed using a single-item measure [30], rated by a 5-point Likert scale. Participants were provided with the following definition: “Stress means a situation in which a person feels tense, restless, nervous, or anxious or is unable to sleep at night because his/her mind is troubled all the time” and asked, “Do you feel this kind of stress these days?” The scale ranged from 1 (not at all) to 5 (very much), where a higher score indicates greater levels of stress that individuals are experiencing. This method is associated with cortisol secretion [31], a key biomarker used for assessing psychological stress levels, demonstrating its sensitivity in stress assessment. Its applicability and robustness have been further evidenced in the Chinese workplace setting [32, 33].

### Demographic, lifestyle, and occupational characteristics

Based on previous empirical studies and systematic reviews [34–36], potential covariates were identified when examining the association between sedentary behaviour and mental health outcomes, including sociodemographic and lifestyle factors.

#### Sociodemographic characteristics

To mitigate potential participant discomfort regarding sensitive information, an “I prefer not to answer” option was provided for all demographic questions.

##### Age group

Age was divided into six groups, i.e., “18–24”, “25–34”, “35–44”, “45–54”, “55–64”, and “65 and over”. Since no participants chose the “55–64” and “65 and over” options, these two groups were merged into “55 and over” for data analysis and reporting.

##### Sex

Measured as “male” and “female”.

##### Marital status

Measured using five options: “single,” “married,” “separated,” “divorced,” and “widowed.” This variable was subsequently converted into a dummy variable for data analysis and reported as “married” and “non-married.”

##### Educational level

Education was measured using the following categories: “primary school or below,” “middle school,” “senior high school/secondary vocational school,” “undergraduate degree/higher vocational school,” and “master’s degree or above.” The first three categories were merged into “education at or below secondary school” for data analysis and reporting.

##### Body mass index (BMI)

BMI was calculated by self-reported weight and height, using the formula body mass divided by height squared (kg/m^2^).

#### Lifestyle characteristics

##### Smoking status

“Never smoked,” “former smoker,” “current occasional smoker,” and “current daily smoker.” This variable was converted into dummy variables: “never smoked” and “former or current smoker.”

##### Alcohol consumption

The Alcohol Use Disorders Identification Test for Consumption (AUDIT-C) was used [37]. The AUDIT-C consists of the first three questions of the 10-item AUDIT [38] and primarily asks participants about alcohol consumption, including the frequency and amount (e.g., “How often do you have a drink containing alcohol?”). The AUDIT-C has been validated as an efficient drinking screening tool among the Chinese population [36, 39]. Cronbach’s α for the AUDIT-C was 0.839 in this study.

##### Poor sleep quality

The Athens Insomnia Scale (AIS) was employed to examine sleep quality. The AIS is a brief instrument used to evaluate insomnia severity using eight items, each rated from 0 to 3 (e.g., “Sleepiness during the day: None, Mild, Considerable, Intense”) [40]. A higher AIS score indicates poorer sleep quality. A cut-off score of 6 or higher was used to indicate the presence of poor sleep quality [41]. Cronbach’s α for the AIS was 0.839 in this study.

##### Physical activity

Participants complete the short-form IPAQ to estimate physical activity [20]. The IPAQ measures the intensity and duration of physical activity within the past week. It has been used in the Chinese population and has shown acceptable reliability and validity [21, 36, 42].

#### Occupational characteristics

##### Job position

“Ordinary employee,” “frontline manager,” “middle manager,” “senior manager,” and “other.” This variable was converted into a dummy variable: “ordinary employees” and “managerial employees and others.”

##### Duration in the current company (Tenure)

“Less than 1 year,” “1–3 years,” “4–6 years,” “7–9 years,” and “more than 10 years.”

##### Duration in the current industry

“Less than 1 year,” “1–3 years,” “4–6 years,” “7–9 years,” and “more than 10 years.”

##### Workdays per week

Five days, 6 days, or a “big/small week working scheme” (meaning that for two weeks each month, employees work 6 days a week).

##### Daily working minutes

Participants were asked to report their arrival and departure times at the company on a typical workday. These times were then converted into working minutes per day.

##### Job satisfaction

The 6-item Job Satisfaction Index was used to assess job satisfaction (e.g., “How satisfied are you with the nature of the work you perform?”) [43]. The scale demonstrated good internal consistency in this study (Cronbach’s α = 0.933).

### Statistical analysis

For descriptive statistics, Shapiro-Wilk tests were first performed, revealing that most continuous variables were non-normally distributed. Categorical variables were described using frequencies and percentages. For sociodemographic characteristics, means and standard deviations (SD) were used to describe variables with skewness less than 1, while medians and interquartile ranges (IQR) were used for variables with skewness greater than 1. However, to provide a comprehensive description of the distributions for key study variables (i.e., occupational and total sedentary behaviour, depression, anxiety, and stress), both means (SD) and medians (IQR) were reported.

Inferential analysis was conducted in two phases: hierarchical ordinal logistic regression and path analysis.

All analyses were conducted using R (version 4.4.0) with packages MICE [44], dplyr [45], purrr [46], and MASS [47], and SPSS AMOS 28.0.

#### Hierarchical ordinal regression

**Fig. 1:**
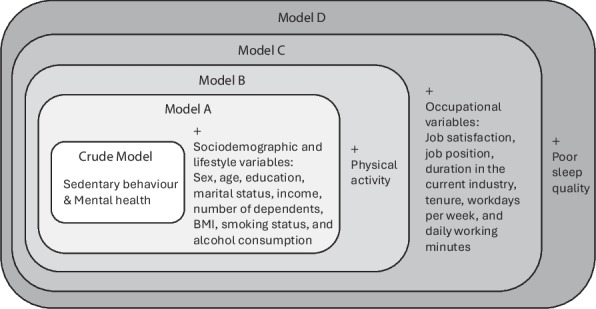
Hierarchical regression modelling strategy. Note. The diagram illustrates the hierarchical regression models, with variables entered sequentially to examine their incremental effects on the associations between sedentary behaviour and mental health outcomes. Crude Model: Unadjusted association between sedentary behaviour (both total and occupational) and mental health outcomes. Model A: Adjusted for sociodemographic and lifestyle variables. Model B: Additionally adjusted for physical activity. Model C: Additionally adjusted for occupational variables. Model D: The final model, further adjusted for poor sleep quality

To examine the robustness of the associations between sedentary behaviour (total and occupational) and mental health symptoms, a five-step hierarchical ordinal regression approach was employed. This strategy allowed for the observation of how associations changed as different clusters of covariates were sequentially introduced [48]. The modelling hierarchy was structured as Fig. 1.

The rationale for this sequencing was theoretically and empirically driven. Model A accounted for baseline sociodemographic and lifestyle confounders [49, 50]. Regarding Model B, physical activity was treated as a distinct component rather than being grouped with initial lifestyle adjustments. While previous research suggests that physical activity may act as an attenuating factor in the association between sedentary behaviour and mental health [51], substantial empirical evidence indicates that sedentary behaviour exerts effects independent of physical activity [52–54]. Consequently, physical activity was included separately in Model B to rigorously test whether the association between sedentary behaviour and mental health remained significant despite this potential attenuation. Occupational factors were added in Model C to assess whether the context of sedentary behaviour influences the association [55, 56]. Finally, poor sleep quality was entered in Model D. Although often classified as a lifestyle variable, evidence from physical activity studies identifies sleep as a potential mediator rather than a simple confounder [57, 58]. Therefore, sleep quality was introduced in the final stage to prevent overadjustment that could obscure the effects of preceding variables [59, 60].

Prior to analysis, sedentary behaviour variables were rescaled from minutes to hours to enhance interpretability (i.e., estimates represent risk per 60-minute increase). Results are presented as Odds Ratios (OR) with 95% Confidence Intervals (CI).

Multicollinearity was assessed using the variance inflation factor (VIF), with VIF values of 5.0 or greater indicating a potential multicollinearity problem.

#### Path analysis

Following hierarchical regression analyses, path analysis was employed to address the limitations of standard regression in examining complex interrelationships among multiple variables. Path analysis allows multiple associations to be examined simultaneously within a single model [61], facilitating the exploration of hypothetical direct and indirect cross-sectional associations between occupational sedentary behaviour and mental health outcomes.

**Fig. 2:**
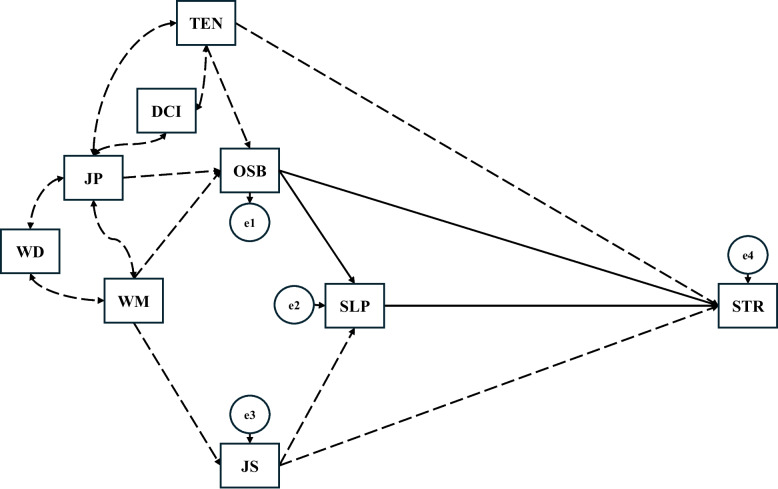
Hypothesised path analysis model. Note. OSB=occupational sedentary behaviour, SLP=poor sleep quality, STR=stress, TEN=tenure, DCI=duration in current industry, JP=job position, WD=workdays per week, WM=daily working minutes, JS=job satisfaction. Solid arrows represent a priori theoretical paths; dashed arrows represent exploratory paths. Arrows indicate the hypothesised direction of associations specified for the regression model and do not imply proven causal relationships in this cross-sectional design. A double-headed arrow represents a correlation

The proposed conceptual model (Fig. 2) was specified based on a combination of prior theoretical evidence and exploratory analyses [62], and is explicitly intended to represent a hypothetical, rather than causal, cross-sectional association structure.

1. Pre-specified Theoretical Paths (Solid Arrows): Based on previous evidence suggesting that poor sleep quality may mediate the association between physical activity and mental health [57, 58], this study a priori hypothesised that occupational sedentary behaviour might be indirectly associated with stress through poor sleep quality. To preliminarily evaluate this, Baron and Kenny’s four-step approach was applied [63], confirming that criteria for mediation testing were satisfied. This component was therefore theory-informed and pre-specified.
2. Exploratory Paths (Dashed Arrows): In contrast, evidence regarding the roles of occupational characteristics in these associations remains limited. Consequently, an abductive approach was adopted, in which exploratory regression analyses were used to infer plausible associations for model specification rather than for formal theory testing [62]. Two complementary approaches were used: single-factor models and sequentially adjusted models (Supplementary document 1). These paths were treated as empirically identified and exploratory, given the absence of strong a priori hypotheses.

The final path model (Fig. 2) visually distinguishes between these pre-specified (solid lines) and exploratory (dashed lines) components. Paths involving occupational characteristics should be interpreted with caution due to the risk of overfitting inherent in exploratory specification.

While acknowledging the bidirectional nature of the relationship between sedentary behaviour and mental health, this study prioritised investigating the directional influence of occupational sedentary behaviour on mental health outcomes. This specification is based on the premise that occupational behaviour is a modifiable lifestyle factor, serving as a target for intervention. Therefore, arrows in the model represent the hypothesised direction of association for statistical estimation, rather than asserting established causality.

Spearman’s rank correlations were calculated for all modelled variables. Path analysis was performed using Maximum Likelihood Estimation (MLE) in Amos to estimate standardised regression weights. Bootstrapping (5000 iterations) was used to estimate potential indirect effects and confidence intervals, and to address non-normal data distribution [64]. Model fit was assessed using established indices [65]: Chi-square to degrees of freedom ratio (Chi/df) ≤ 3, goodness-of-fit index (GFI) ≥ 0.90, Comparative Fit Index (CFI) ≥ 0.95, Tucker-Lewis Index (TLI) ≥ 0.95, and Root Mean Square Error of Approximation (RMSEA) ≤ 0.06. Additionally, the Akaike Information Criterion (AIC) and Bayesian Information Criterion (BIC) were employed for model comparison and selection, with lower values indicating a superior and more parsimonious model.

To ensure robust results, the analysis was conducted based on pooled estimates derived from five imputed datasets (as detailed in Section “Missing data and sensitivity analysis”). The full hypothetical model was initially evaluated. Subsequently, to obtain a parsimonious model, non-significant paths were sequentially removed based on statistical significance and improvements in model fit indices (e.g., reductions in AIC and BIC).

#### Missing data and sensitivity analysis

Prior to analysis, missing data were imputed using multiple imputation with the random forest algorithm via RStudio. Five imputed datasets were generated. These datasets were subsequently analysed in parallel for hierarchical regression and correlation analyses, with final results pooled according to Rubin’s rules [66]. For the path analysis, a hybrid reporting strategy was adopted. Model fit indices were pooled by calculating the arithmetic mean across the five imputed datasets [67]. For parameter estimates (i.e., path coefficients), the results from the first imputed dataset are presented as the primary model to ensure the interpretability of significance tests. Significance testing was based on unstandardised regression coefficients, while standardised coefficients are reported to facilitate the comparison of effect sizes. To ensure the robustness and reliability of these findings, a sensitivity analysis was conducted by running the model across the remaining four imputed datasets. Comparison across all five datasets confirmed high consistency in model fit indices and parameter estimates (Supplementary document 2).

## Results

### Participants

Four organisations participated in this study, all of which were technology-driven private enterprises located in Wuhan, China. One organisation employed fewer than 500 staff, while the remaining three had between 500 and 1,000 employees. Across all participating organisations, the workforce was primarily office-based, with Software and IT professionals constituting approximately 50–70% of employees.

The survey received 322 responses from employees across four companies, resulting in a response rate of approximately 27%. Among these, 235 participants fully completed the survey, with no missing data on occupational sedentary behaviour or mental health symptoms.

### Descriptive data

Descriptive statistics, as well as missing data for the sample, are presented in Tables 1, 2 and 3. Most participants were male (68.1%), aged 25–34 (60.9%), had received tertiary education (92.3%), and were non-married (56.2%). The largest proportion of the sample (31.5%) reported an annual income of CNY 80,000–140,000, and 41.7% reported having no dependents. Participants reported an average actual working time of 590.8 (± 68.7) minutes (≈ 9.85 ± 1.15 hours) per workday. Within this time, the mean occupational sedentary behaviour was 427.9 (± 133.2) minutes (≈ 7.13 ± 2.22 hours), and the total daily sedentary behaviour was 499.9 (±161) minutes (≈ 8.33 ± 2.68 hours). According to these self-reported measures, occupational sedentary time accounted for 72.4% of working hours, which equated to 347.52 minutes (≈ 5.79 hours) in a standard 8-hour working day.

**Table 1:** Demographic characteristics (*N*=235)

**Variables**	**Frequency**
Sex, n (%)	
Male	160 (68.1)
Female	73 (31.1)
Data not reported	2 (0.9)
Age, n (%)	
18–24	56 (23.8)
25–34	143 (60.9)
35–44	31 (13.2)
45–54	2 (0.9)
Over 55	0 (0)
Data not reported	3 (1.3)
Educational level, n (%)	
At or below secondary school	2 (0.9)
Undergraduate degree/Higher vocational school	217 (92.3)
Master’s degree or above	12 (5.1)
Data not reported	4 (1.7)
Annual income, n (%)	
Less than 80000 CNY	66 (28.1)
80000–140000 CNY	74 (31.5)
140000–190000 CNY	25 (10.5)
200000–250000 CNY	11 (4.7)
More than 250000 CNY	12 (5.1)
Data not reported	47 (20.0)
Marital status, n (%)	
Married	80 (34)
Non-married	132 (56.2)
Data not reported	23 (9.8)
Number of dependents, n (%)	
0	98 (41.7)
1	28 (11.9)
2	34 (14.5)
3	18 (7.7)
More than 4	21 (8.9)
Data not reported	36 (15.3)

**Table 2:** Sedentary behaviour and mental health (*N*=235)

**Variables**	**Frequency**	**Mean/Median**
Physical activity (METs), median (25%–75%)		612 (109.5–1923.8)
Occupational sedentary behaviour (min), mean (SD)/median (25%–75%)		427.9 (±133.2)/480 (360–480)
Total sedentary behaviour (min), mean (SD)/median (25%–75%)		499.9 (±161.0)/480 (420–600)
Depression, mean (SD)/median (25%–75%)		7.5 (±3.8)/8 (5–10)
No/Low symptom scores	111 (47.2)	
Mild symptom scores	76 (32.3)	
Moderate to Severe symptom scores	48 (20.4)	
Anxiety, mean (SD)/median (25%–75%)		7.6 (±3.9)/8 (5–10)
No/Low symptom scores	114 (48.5)	
Mild symptom scores	70 (29.8)	
Moderate to Severe symptom scores	51 (21.7)	
Stress, mean (SD)/median (25%–75%)		2.3 (±0.9)/2 (2–3)

Missing data of continuous variables: physical activity (6.8%), and self-reported total sedentary behaviour (7.7%)

**Table 3:** Lifestyle and occupational characteristics (*N*=235)

**Variables**	**Frequency**	**Mean/Median**
Smoking status, n (%)		
Never smoked	151 (64.3)	
Former or current smoker	84 (35.7)	
AUDIT-C, median (25%–75%)		1 (1–3)
BMI, median (25%–75%)		23.5 (21.1–28.0)
Sleep, mean (SD)		6.0 (±4.0)
Job position, n (%)		
Ordinary employees	194 (82.6)	
Managerial employees and other	41 (17.4)	
Daily working minutes, mean (SD)		590.8 (±68.7)
Workdays per week, n (%)		
5	199 (84.7)	
6	16 (6.8)	
Big/small week scheme (employees work a six-day week every second week)	20 (8.5)	
Duration in the current industry (year), n (%)		
Less than 1	28 (11.9)	
1–3	81 (34.5)	
4–6	54 (23.0)	
7–9	38 (16.2)	
More than 10	34 (14.5)	
Tenure (year), n (%)		
Less than 1	55 (23.4)	
1–3	117 (49.8)	
4–6	38 (16.2)	
7–9	12 (5.1)	
More than 10	13 (5.5)	
Job satisfaction, mean (SD)		22.3 (±5.9)

Missing data of continuous variables: alcohol (20%), BMI (3.8%), sleep quality (0.4%), and daily working minutes (0.4%)

Participants reported moderate mean scores for both depression and anxiety symptoms, as assessed by the Hospital Anxiety and Depression Scale (HADS). The average scores for depression and anxiety were 7.5 (±3.8) and 7.6 (±3.9), respectively. Further analysis using the established HADS cut-offs (0–7: No/Low symptom scores; 8–10: Mild symptom scores; ≥11: Moderate to Severe symptom scores) indicated that a considerable proportion of participants exhibited elevated symptom levels. Specifically, 20.4% (*n* = 48) had depression scores and 21.7% (*n* = 51) had anxiety scores falling within the range corresponding to moderate to severe symptom levels. The mean stress score was 2.3 (±0.9) on a 1–5 scale. This value lies below the scale’s mid-point of 3.

### Association of sedentary behaviour and mental health

The association of total and occupational sedentary behaviour and symptoms of depression, anxiety, and stress are presented separately in Table 4. The variance inflation factor (VIF) analysis revealed that all VIF values were below 3, indicating no multicollinearity in this study.

**Table 4:** Association of sedentary behaviour and mental health symptoms indicators

	**Depression**	**Anxiety**	**Stress**
	OR (95% CI)	OR (95% CI)	OR (95% CI)
Association of occupational sedentary behaviour and mental health symptoms			
Crude	1.08 (0.95, 1.22)	1.09 (0.96, 1.23)	1.13^*^ (1.01, 1.26)
A	1.08 (0.95, 1.23)	1.09 (0.96, 1.24)	1.12^*^ (1.00, 1.26)
B	1.09 (0.96, 1.25)	1.09 (0.96, 1.25)	1.12^*^ (1.00, 1.26)
C	1.02 (0.88, 1.19)	1.04 (0.90, 1.21)	1.09 (0.96, 1.23)
D	1.00 (0.85, 1.17)	1.01 (0.85, 1.19)	1.04 (0.92, 1.18)
Association of total sedentary behaviour and mental health symptoms			
Crude	1.06 (0.95, 1.19)	1.10 (0.98, 1.23)	1.17^**^ (1.05, 1.30)
A	1.07 (0.95, 1.21)	1.10 (0.98, 1.24)	1.20^**^ (1.08, 1.35)
B	1.07 (0.95, 1.21)	1.10 (0.98, 1.24)	1.20^**^ (1.08, 1.35)
C	1.03 (0.91, 1.18)	1.08 (0.95, 1.23)	1.19^**^ (1.06, 1.34)
D	1.00 (0.87, 1.15)	1.03 (0.89, 1.20)	1.15^*^ (1.02, 1.29)

Model A= Crude model plus sex, age, education, marital status, income, number of dependents, BMI, smoking status, and alcohol consumption. Model B= Model A plus physical activity. Model C= Model B plus job satisfaction, job position, duration in the current industry, tenure, workdays per week, and daily working minutes. Model D= Model C plus sleep

*OSB* occupational sedentary behaviour, *TSB* total sedentary behaviour, *DEP* depression, *ANX* anxiety, *STR* stress

^*^p ≤ 0.05, ^**^p ≤ 0.01, ^***^p ≤ 0.001

#### Total sedentary behaviour and symptoms of depression, anxiety, and stress

No significant association was observed between total sedentary behaviour and symptoms of depression (*OR* = 1, 95% *CI*: 0.87–1.15; *p* = 0.97) or anxiety (*OR* = 1.03, 95% *CI*: 0.89–1.2; *p* = 0.66) across all models. However, in the fully adjusted models, total daily sitting time showed a consistent, significant association with higher stress (*OR* = 1.15, 95% *CI*: 1.02–1.29; *p* = 0.02). This indicates that each additional hour of daily total sedentary behaviour is associated with 15% higher odds of reporting a more severe category of stress.

#### Occupational sedentary behaviour and symptoms of depression, anxiety, and stress

Occupational sedentary behaviour was not significantly associated with depression (*OR* = 1, 95% *CI*: 0.85–1.17; *p* = 0.99) or anxiety (*OR* = 1.01, 95% *CI*: 0.85–1.19; *p* = 0.94). Regarding stress, significant associations were observed in the crude model (*OR* = 1.13, 95% *CI*: 1.01–1.26; *p* = 0.03), Model A (*OR* = 1.12, 95% *CI*: 1–1.26; *p* = 0.04), and Model B (*OR* = 1.12, 95% *CI*: 1–1.26; *p* = 0.04). However, this association was attenuated and lost statistical significance in Model C (*OR* = 1.09, 95% *CI*: 0.96–1.23; *p* = 0.19) and the fully adjusted model D (*OR* = 1.04, 95% *CI*: 0.92–1.18; *p* = 0.53), after adjusting for occupational variables and sleep quality.

### Correlations

Prior to conducting path analysis, pairwise correlations among included variables were assessed to detect potential multicollinearity (see Table 5) [68]. Occupational sedentary behaviour was positively correlated with the daily working minutes (*r* = 0.255, *p* ≤ 0.001) and stress (*r* = 0.185, *p* = 0.005). A longer tenure was associated with a reduction in occupational sedentary time (*r* = −0.195, *p* = 0.003). Poor sleep quality was positively correlated with stress (*r* = 0.374, *p* ≤ 0.001). Tenure was positively correlated with duration in the current industry (*r* = 0.620, *p* ≤ 0.001). Managerial jobs showed fewer working minutes per day (*r* = −0.131, *p* = 0.046), less time in occupational sedentary behaviour (*r* = 0.237, *p* ≤ 0.001), fewer working days per week (*r* = −0.210, *p* ≤ 0.001), longer duration in their current company (*r* = −0.410, *p* ≤ 0.001) and industry (*r* = −0.396, *p* ≤ 0.001) than ordinary job positions. The number of working days per week was positively correlated with daily working minutes (*r* = 0.186, *p* = 0.004). Daily working minutes were positively correlated with job satisfaction (*r* = 0.138, *p* = 0.034). Finally, job satisfaction was negatively correlated with poor sleep quality (*r* = −0.210, *p* ≤ 0.001).

**Table 5:** Correlations between variables included in the path analysis

Variables	1	2	3	4	5	6	7	8	9
1. OSB	-								
2. SLP	0.089	-							
3. STR	0.185^**^	0.374^***^	-						
4. TEN	−0.195^**^	−0.046	−0.119	-					
5. DCI	−0.136^*^	−0.047	−0.024	0.620^***^	-				
6. JP	0.237^***^	0.073	0.073	−0.410^***^	−0.396^***^	-			
7. WD	−0.006	0.073	0.097	0.084	0.113	−0.210^***^	-		
8. WM	0.255^***^	−0.023	0.068	0.083	−0.002	−0.131^*^	0.186^**^	-	
9. JS	0.005	−0.210^***^	−0.117	0.034	0.157*	−0.083	0.013	0.138^*^	-

*OSB* Occupational sedentary behaviour, *SLP* Poor sleep quality, *STR* Stress, *TEN* Tenure, *DCI* Duration in current industry, *JP* Job position, *WD* Workdays per week, *WM* Daily working minutes, *JS* Job satisfaction

^*^p ≤ 0.05, ^**^p ≤ 0.01, ^***^p ≤ 0.001

Although many of the correlations were statistically significant, the coefficients were relatively small (e.g., *r* = 0.1–0.3). This pattern suggests that multicollinearity is not a major concern, supporting the use of path analysis to explore their underlying latent structure [69].

### Path analysis

The initial proposed model showed adequate fit (see Supplementary document 2 for full model comparison). However, analysis revealed that paths associated with two specific occupational characteristics (i.e., workdays per week and duration in current industry) were not statistically significant (*p* > 0.05). Therefore, these non-significant paths were trimmed. The final model demonstrated improved mean fit indices (i.e., Chi/df = 0.501, GFI = 0.994, CFI = 1.000, TLI = 1.076, and RMSEA = 0.000). Notably, the mean AIC decreased from 74.618 to 42.507, and the mean BIC decreased from 161.108 to 108.240, indicating a more parsimonious and robust model structure.

**Fig. 3:**
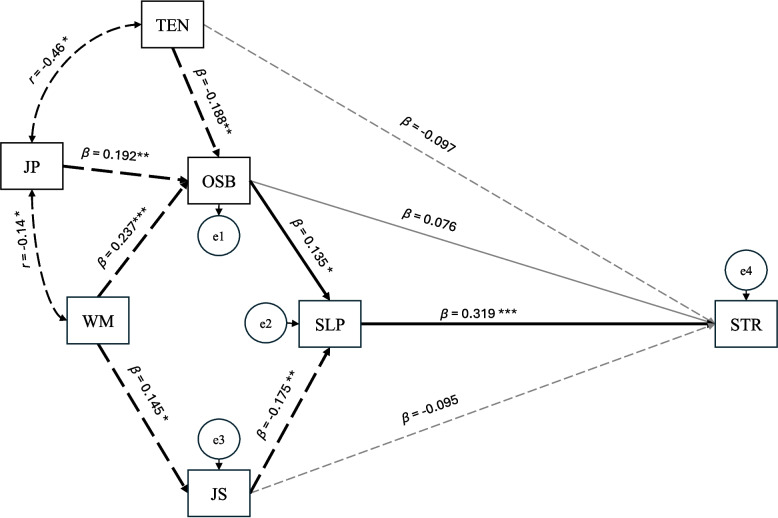
Path analysis results. Note. OSB=occupational sedentary behaviour, SLP=poor sleep quality, STR=stress, TEN=tenure, JP=job position, WM=daily working minutes, JS=job satisfaction. Solid arrows represent a priori theoretical paths; dashed arrows represent exploratory paths. Significant paths are highlighted in bold black lines, while non-significant paths are displayed as thin grey lines. Arrows indicate the hypothesised direction of associations and do not imply proven causal relationships. Values represent standardised estimates from the first imputed dataset: standardised path coefficients (*β*) for single-headed arrows and correlations (*r*) for double-headed arrows. Significance: *p ≤ 0.05, **p ≤ 0.01, ***p ≤ 0.001

As presented in Figure 3 and Table 6, the path analysis revealed that occupational sedentary behaviour was not associated with stress (*β* = 0.076, *p* = 0.231). Occupational sedentary behaviour was positively associated with poor sleep quality (i.e., longer sedentary time at work was linked to worse sleep quality) (*β* = 0.135, *p* = 0.035). Lower job satisfaction was associated with poorer sleep quality (i.e., higher job satisfaction was associated to better sleep quality) (*β* = −0.175, *p* = 0.006). Poor sleep quality, in turn, was positively associated with higher levels of stress (*β* = 0.319, *p* < 0.001). Higher daily working time was associated with higher levels of job satisfaction (*β* = 0.145, *p* = 0.025). Daily working time, job position, and tenure were antecedent variables of occupational sedentary behaviour, indicating that: 1) longer working time were associated with higher occupational sedentary behaviour (*β* = 0.237, *p* < 0.001); 2) non-managerial employees spent more time on occupational sedentary behaviour than managerial employees (*β* = 0.192, *p* = 0.005); and 3) employees with longer tenure in the company tended to spend less time in occupational sedentary behaviour (*β* = −0.188, *p* = 0.006).

**Table 6:** Unstandardised and standardised path coefficients from the trimmed path model

**Estimator**	***β***	***b***	***S.E.***
Outcome: Occupational sedentary behaviour			
Daily working minutes	0.237	0.462^***^	0.119
Job position	0.192	68.364^**^	24.342
Tenure	−0.188	−24.456^**^	8.832
Outcome: Sleep quality			
Occupational sedentary behaviour	0.135	0.004^*^	0.002
Job satisfaction	−0.175	−0.119^**^	0.043
Outcome: Stress			
Occupational sedentary behaviour	0.076	0.001	0.000
Job satisfaction	−0.095	−0.015	0.010
Sleep quality	0.319	0.074^***^	0.014
Tenure	−0.097	−0.086	0.056
Outcome: Job satisfaction			
Daily working minutes	0.145	0.012^*^	0.006

Values represent standardised coefficients (*β*) from the first imputed dataset. Significance testing was based on unstandardised regression coefficients; standardised coefficients are reported for comparison of effect sizes

*β* Standardised coefficient, *b* Unstandardised regression coefficient, *S.E.* Standard error

^*^p ≤ 0.05, ^**^p ≤ 0.01, ^***^p ≤ 0.001

The VIF analysis confirmed the absence of multicollinearity between tenure and occupational sedentary behaviour.

Mediation analysis of poor sleep quality between occupational sedentary behaviour and stress showed that occupational sedentary behaviour was not directly (*β* = 0.119, *p* = 0.276) or indirectly (*β* = 0.043, *p* = 0.059) associated with stress (Table 7). Direct and indirect effects of other variables on stress were examined. Job satisfaction was found to be negatively associated with stress levels when mediated by sleep quality (*β* = −0.056, *p* = 0.015).

**Table 7:** Standardised direct, indirect, and total effects of included variables on stress

**Path**	**Direct Effects**	**Indirect Effects**	**Total Effects**
Effects of occupational sedentary behaviour on stress via sleep quality			
OSB→SLP→STR	0.076	0.043	0.119
Effects of other variables on stress			
SLP→STR	0.319^***^	-	0.319^***^
JS→STR	−0.095	−0.056^*^	−0.151^*^
TEN→STR	−0.097	−0.022	−0.119
JP→STR	-	0.023	0.023
WM→STR	-	0.006	0.006

Values represent standardised coefficients (*β*) from the first imputed dataset. Significance levels for direct effects were determined based on unstandardised regression coefficients (*b*).

*OSB* Occupational sedentary behaviour, *SLP* Sleep quality, *STR* Stress, *TEN* Tenure, *DCI* Duration in current industry, *JP* Job position, *WD* Workdays per week, *WM* Daily working minutes, *JS* Job satisfaction

^*^p ≤ 0.05, ^**^p ≤ 0.01, ^***^p ≤ 0.001

## Discussion

This study provided an overview of occupational sedentary behaviour and mental health symptoms among Software and IT employees in China through an online survey. It aimed to 1) estimate the duration of sedentary behaviour and the level of mental health symptoms among software and IT workers in China, 2) determine whether there is an association between occupational sedentary behaviour and common mental health symptoms, and 3) identify variables that may influence the association between occupational sedentary behaviour and common mental health symptoms.

### Duration of sedentary behaviour and the level of mental health symptoms

The Software and IT workers in this study engaged in a high volume of occupational sedentary behaviour. The mean occupational sedentary behaviour was 427.9 (± 133.2) minutes (7.13 ± 2.22 hours), and total daily sedentary behaviour was 499.9 (± 161) minutes (8.33 ± 2.68 hours) during the workday. Occupational sedentary time accounted for 72.4% of working hours, which equated to 347.52 minutes (5.79 hours) in a standard 8-hour working day. This self-reported proportion is comparable to device-based observations among employees in the UK (72.6%) [70]. Although slightly lower than device-measured sedentary time in some office, customer service, and call centre settings (77–81.8%) [14, 71, 72], it unsurprisingly remains substantially higher than in physically active occupations such as construction work, where sedentary time accounts for less than 50% of working hours [73].

The average score for depression, anxiety, and stress among Software and IT workers was 7.5 (±3.8), 7.6 (±3.9), and 2.3 (±0.9), respectively. While both mean scores for depression and anxiety fall below the clinical cutoff of 8 (scores are considered normal) [26], the proximity of these mean scores to the threshold is critical, as it indicates that some participants likely exhibit subclinical symptoms. Conversely, participants reported a low average stress score of 2.3 on the 1–5 scale, indicating participants generally experienced low to moderate levels of stress compared to the scale’s mid-point of 3. This discrepancy may be partly due to the single-item stress measure, which might underestimate stress levels by capturing only a general impression rather than the multidimensional aspects of stress. Alternatively, this pattern may potentially be explained by a state of emotional exhaustion or burnout, where individuals no longer perceive acute stress but still experience significant emotional symptoms [74, 75]. However, further evaluation is needed for this explanation.

The observed proportion of participants with moderate to severe levels of depression and anxiety symptoms (20.4% and 21.7%, respectively) was higher than the estimated prevalence of clinically diagnosed mental health disorders in the general population in China (16.6%) [18]. This difference should not be interpreted as a higher clinical incidence among the current population, as the measure in this study reflects self-reported symptoms at the time of data collection rather than confirmed diagnoses. However, this finding suggests that employees in the Software and IT sector may be at elevated risk of mental distress.

Overall, the occurrence of high occupational sedentary behaviour and pre-clinical mental health scores is a critical finding, indicating that Software and IT workers in China require targeted interventions to reduce occupational sedentary behaviour and promote mental health.

### Association between occupational sedentary behaviour and mental health symptoms

Previous systematic reviews have suggested that higher volumes of sedentary behaviour are associated with increased risks of depression and anxiety [4, 76]. In contrast, no statistically significant associations were observed between occupational sedentary behaviour and these mental health outcomes in the current study. This discrepancy may be related to the specific characteristics of the study sample and the contextual nature of sedentary behaviour.

Specifically, unlike previous studies that encompassed broader demographic groups and predominantly passive sedentary contexts, such as television viewing among older adults [77], the present sample consisted of young Software and IT professionals for whom work-related sitting was the dominant sedentary context. It is plausible that the occupational characteristics of IT work (e.g. higher levels of cognitive engagement, creativity, and job control) may attenuate the potential adverse psychological effects of sedentary behaviour. These features have been suggested to buffer psychological distress and may differentiate occupational sedentary behaviour from more passive forms of sedentary behaviour [49]. Consequently, the mental health implications of sedentary behaviour in this occupational group may differ from those observed in other populations or occupational settings [55, 78, 79].

With regard to stress, a divergence was observed between total and occupational sedentary behaviour. While total sedentary behaviour remained consistently associated with stress across models, the association between occupational sedentary behaviour and stress was attenuated and became non-significant after adjustment for occupational variables and sleep quality. This pattern suggests that the relationship between workplace sitting and stress may be indirect and influenced by intermediate factors rather than reflecting a direct effect. Although sedentary behaviour has been linked to physiological stress responses, such as alterations in cortisol levels [54], the present findings indicate that, within an occupational context, these associations may be contingent upon broader work-related and behavioural factors, particularly sleep quality, which is explored further in the subsequent section.

### Influencing factors on the association

Occupational sedentary behaviour initially demonstrated a statistically significant association with stress; however, this association was attenuated and became non-significant after adjustment for occupational variables (Model C) and sleep quality (Model D). This pattern suggests that the relationship between occupational sedentary behaviour and stress is unlikely to be direct and may be shaped by work-related and sleep factors.

To further examine the structure of these cross-sectional associations, path analysis was conducted as an exploratory, theory-informed approach. Sleep quality was specified as a hypothesised intermediary factor. The model suggested a plausible indirect pattern, with longer occupational sedentary time associated with poorer sleep quality, which in turn was associated with higher stress levels. Mechanistically, prolonged occupational sitting, often coupled with extended screen exposure, may disrupt circadian rhythms or result in insufficient physical fatigue to initiate restorative sleep [80]. Poor sleep, in turn, impairs emotional regulation and cognitive coping resources, thereby increasing susceptibility to stress [81]. This hypothesised pattern is consistent with previous literature suggesting that sleep may play an intermediary role in the association between movement-related behaviours and mental health outcomes [57, 58].

Statistically, however, the estimated indirect association did not reach conventional significance in current study (*β* = 0.043, *p* = 0.059). Several factors may account for this non-significance. First, the current sample size (N = 235) may have limited statistical power to detect small indirect associations, which typically require larger samples than direct associations. Second, measurement error, particularly in the assessment of stress using subjective self-reported measures, may have attenuated the observed associations. Finally, the non-significant result may reflect a genuinely weak association. Supporting this interpretation, a previous large-scale study involving 1,843 participants reported a similarly small effect size for the association between sedentary behaviour and mental health [78]. Taken together, these findings suggest that any association between occupational sedentary behaviour and stress is likely to be modest and indirect, rather than reflecting a robust direct relationship. Future research with larger samples and more objective measures is warranted to further explore these exploratory associations.

Tenure was also identified as a potential influential factor associated with both occupational sedentary behaviour and stress. In exploratory single-factor regressions, longer tenure was negatively associated with occupational sedentary behaviour and stress, while occupational sedentary behaviour was positively associated with stress. When tenure and occupational sedentary behaviour were included concurrently in a path model, neither variable showed a statistically significant association with stress. This attenuation may be attributable to the small effect sizes of both variables and the reduced statistical power associated with modelling them simultaneously [82]. In addition, the opposing directions of the associations between tenure and occupational sedentary behaviour with stress may have partially offset one another [48].

Although the direct association between tenure and stress was not retained in the multivariable model, longer tenure remained associated with lower occupational sedentary time, which is consistent with previous research [72]. This pattern may reflect greater job familiarity, increased task variation, or enhanced autonomy over work routines among more experienced employees.

From a practical perspective, these findings highlight the need for more tailored workplace interventions. Specifically, integrating non-sedentary practices (e.g., standing meetings or scheduled active breaks) into onboarding programmes may be particularly beneficial for new employees, as this approach helps establish healthier behavioural norms early in their tenure. Furthermore, given the potential intermediary role of sleep identified in the path analysis, multifaceted workplace wellness programmes may be warranted. Such programmes should extend beyond physical activity alone to incorporate sleep hygiene education and strategies aimed at enhancing job satisfaction, thereby supporting the holistic well-being of Software and IT professionals.

### Strengths and limitations

This study makes several original contributions to the literature on sedentary behaviour and mental health. First, it focuses on a relevant yet understudied occupational group, Software and IT workers, among whom occupational sedentary behaviour constitutes a dominant exposure. Second, the study addresses a contemporary research question with clear practical relevance, particularly in relation to workplace health management.

Methodologically, the use of path analysis allowed for the simultaneous examination of direct and indirect relationships between occupational sedentary behaviour, psychosocial factors, sleep quality, and mental health symptoms, providing a more integrated analytical perspective than traditional regression approaches. The study also demonstrates transparency in handling missing data, with clearly reported imputation procedures.

From a theoretical perspective, the findings contribute to the understanding of psychosocial pathways linking occupational sedentary behaviour and stress-related outcomes, highlighting the potential role of poor sleep quality as an intermediary factor. Finally, the results have practical implications for employers, suggesting that employees with shorter tenure may be particularly vulnerable to prolonged occupational sitting and elevated stress.

However, several limitations exist. First, the use of convenience sampling, combined with the modest sample size and response rate, limits generalisability. This approach risks selection bias, potentially over-representing health-conscious or highly stressed employees. Thus, findings are primarily applicable to similar enterprises in Wuhan rather than the broader Chinese Software and IT workforce. Future research should employ multi-centre, stratified random sampling to enhance representativeness.

Second, the use of a single-item stress measure constitutes a core limitation, though it was chosen to prioritise brevity and minimise questionnaire fatigue. While such measures correlate with biomarkers like cortisol [31], they lack the reliability and validity of multi-item scales and fail to capture the multidimensional nature of stress (e.g., work-related vs. general). This crude categorisation may explain the relatively low stress scores and distinct association patterns observed. Consequently, stress-related findings should be interpreted with caution. Future research should utilise validated multi-dimensional instruments, such as the Perceived Stress Scale (PSS) [83].

Third, it is important to acknowledge the data-driven nature of the proposed model. Given the lack of established theoretical frameworks describing how occupational variables influence the relationship between sedentary behaviour and mental health, this approach was necessary to identify potential associations. Nonetheless, this post hoc model refinement indicates that the findings should be interpreted as exploratory and hypothesis-generating. Crucially, future research is recommended to validate this refined model structure in an independent sample to ensure generalisability.

Fourth, the cross-sectional design precludes the determination of causal relationships. While our model focused on the path from occupational sedentary behaviour to mental health outcomes based on theoretical hypotheses, reverse causality cannot be ruled out (e.g., high mental health symptoms levels leading to increased sedentary time). Therefore, the directionality proposed in this study should be interpreted as a theoretical association rather than a confirmed causal sequence. Longitudinal designs are essential to firmly establish the temporal ordering of these variables, and intervention studies are needed to confirm causal relationships.

Finally, the reliance on self-reported data (IPAQ) introduces potential susceptibility to recall and social desirability biases [84], which may affect measurement accuracy. While the IPAQ is a widely used instrument, it lacks the precision of objective monitoring. To address this, future research should incorporate objective, device-based assessments (e.g., accelerometers) of total and occupational sedentary time to validate these findings.

## Conclusion

This study identified high levels of occupational sedentary behaviour and pre-clinical mental health scores among Software and IT workers in China. No direct associations were identified between occupational sedentary behaviour and depression, anxiety, or stress in this cross-sectional sample. However, a potential indirect cross-sectional association with stress via poor sleep quality was indicated, although this indirect effect did not reach conventional statistical significance. These findings are exploratory and hypothesis-generating rather than causal, and may help inform future longitudinal research and workplace initiatives focusing on sleep hygiene and holistic well-being.

## Supplementary Information

Supplementary Material 1.

Supplementary Material 2.

## Data Availability

Data and code will be available upon reasonable request by emailing the corresponding author.

## Abbreviations

IT: Information Technology
CI: Confidence Interval
VIF: Variance Inflation Factor
OSB: Occupational Sedentary Behaviour
SLP: Poor Sleep Quality
STR: Stress
TEN: Tenure
DCI: Duration in Current Industry
JP: Job Position
WD: Workdays Per Week
WM: Daily Working Minutes
JS: Job Satisfaction
