# Supplementary material for: Occupational sedentary behaviour and mental health symptoms among software and IT workers in China: a cross-sectional study using path analysis
Source: BMC Public Health. 2026 Apr 8;26:1611. doi: 10.1186/s12889-026-26761-2 (PMC13192230; doi:10.1186/s12889-026-26761-2)
Supplement: Supplementary file 1 — Supplementary Material 1. [file 12889_2026_26761_MOESM1_ESM.docx]

**Exploratory Regression for Path Analysis Model Proposal**

1. **Single-factor regressions**

**Table 1**

Single-factor regression between occupational variables and stress

| **Variable** | **β (95% CI)** | ***p* value** |
| --- | --- | --- |
| Job position | 0.35 (-0.27, 0.97) | 0.27 |
| Tenure | -0.26 (-0.49, -0.03) | 0.03 |
| Duration in the current industry | -0.05 (-0.24, 0.14) | 0.60 |
| Workdays per week | 0.37 (-0.05, 0.80) | 0.08 |
| Daily working minutes | 0.00 (0.00, 0.01) | 0.36 |
| Job satisfaction | -0.04 (-0.09, 0.00) |  |

**Table 1**

Single-factor regression between occupational variables and occupational sedentary behaviour

| **Variable** | **β (95% CI)** | ***p* value** |
| --- | --- | --- |
| Job position | 84.19 (40.53, 127.85) | 0.00 |
| Tenure | -33.46 (-49.48, -17.45) | 0.00 |
| Duration in the current industry | -14.85 (-28.45, -1.24) | 0.03 |
| Workdays per week | 10.88 (-19.83, 41.58) | 0.49 |
| Daily working minutes | 0.37 (0.12, 0.61) | 0.00 |
| Job satisfaction | 0.67 (-2.23, 3.58) | 0.65 |

1. **Investigate whether the association between occupational sedentary behaviour and stress remained robust after controlling for each occupational variable**

**Table 1**

Association between Occupational Sedentary Behaviour, Controlling for Job Position

| **Variable** | **β (95% CI)** | ***p* value** |
| --- | --- | --- |
| Occupational sedentary behaviour | 0.002 (0.000, 0.004) | 0.053 |
| Job position | 0.203 (-0.434, 0.840) | 0.532 |

**Table 2**

Association between Occupational Sedentary Behaviour, Controlling for Tenure

| **Variable** | **β (95% CI)** | ***p* value** |
| --- | --- | --- |
| Occupational sedentary behaviour | 0.002 (0.000, 0.003) | 0.112 |
| Tenure | -0.203 (-0.447, 0.041) | 0.102 |

**Table 3**

Association between Occupational Sedentary Behaviour, Controlling for Duration in the Current Industry

| **Variable** | **β (95% CI)** | ***p* value** |
| --- | --- | --- |
| Occupational sedentary behaviour | 0.002 (0.000, 0.004) | 0.038 |
| Duration in the current industry | -0.017 (-0.213, 0.179) | 0.864 |

**Table 4**

Association between Occupational Sedentary Behaviour, Controlling for Workdays per Week

| **Variable** | **β (95% CI)** | ***p* value** |
| --- | --- | --- |
| Occupational sedentary behaviour | 0.002 (0.000, 0.004) | 0.045 |
| Workdays per week | 0.338 (-0.083, 0.758) | 0.116 |

**Table 5**

Association between Occupational Sedentary Behaviour, Controlling for Daily Working Minutes

| **Variable** | **β (95% CI)** | ***p* value** |
| --- | --- | --- |
| Occupational sedentary behaviour | 0.002 (0.000, 0.004) | 0.050 |
| Daily working minutes | 0.001 (-0.003, 0.004) | 0.709 |

**Table 6**

Association between Occupational Sedentary Behaviour, Controlling for Job Satisfaction

| **Variable** | **β (95% CI)** | ***p* value** |
| --- | --- | --- |
| Occupational sedentary behaviour | 0.003 (0.001, 0.005) | 0.011 |
| Job satisfaction | -3.229 (-5.233, -1.224) | 0.002 |
