# Supplementary material for: Occupational sedentary behaviour and mental health symptoms among software and IT workers in China: a cross-sectional study using path analysis
Source: BMC Public Health. 2026 Apr 8;26:1611. doi: 10.1186/s12889-026-26761-2 (PMC13192230; doi:10.1186/s12889-026-26761-2)
Supplement: Supplementary file 2 — Supplementary Material 2. [file 12889_2026_26761_MOESM2_ESM.docx]

**Comparison of model fit indices for the proposed and trimmed models.**

Table S1 Pooled model fit indices for the proposed and trimmed models across five imputed datasets.

|  | **Proposed Model** | | **Trimmed Model** | | **Threshold** |
| --- | --- | --- | --- | --- | --- |
|  | **Mean** | **Range** | **Mean** | **Range** |  |
| Chi/df | 1.231 | 1.229–0.234 | 0.501 | 0.441–0.532 | ≤ 3.00 |
| GFI | 0.978 | 0.978–0.978 | 0.994 | 0.994–0.995 | ≥0.90 |
| CFI | 0.985 | 0.984–0.985 | 1.000 | 1.000–1.000 | ≥0.95 |
| TLI | 0.972 | 0.972–0.972 | 1.076 | 1.071–1.086 | ≥0.95 |
| RMSEA | 0.0312 | 0.031–0.032 | 0.000 | 0.000–0.000 | ≤0.08 |
| AIC | 74.618 | 74.585–74.69 | 42.507 | 41.966–42.788 | Lower is better |
| BIC | 161.108 | 161.075–161.179 | 108.240 | 107.699–108.52 | Lower is better |

**Note.** Values represent the arithmetic mean of fit indices averaged across five imputed datasets, with the range (minimum – maximum) presented in adjacent columns. Chi/df = Chi-square to degrees of freedom ratio; GFI = Goodness-of-Fit Index; CFI = Comparative Fit Index; TLI = Tucker-Lewis Index; RMSEA = Root Mean Square Error of Approximation; AIC = Akaike Information Criterion; BIC = Bayesian Information Criterion. Lower values for AIC and BIC indicate a better trade-off between model fit and complexity.

**񾉣**

**Path analysis across all imputed datasets**

**Imputed data 2**

Table 6a Unstandardised and standardised path coefficients from the path model

| **Estimator** | ***β*** | ***b*** | ***S.E.*** |
| --- | --- | --- | --- |
| **Outcome: Occupational sedentary behaviour** | | | |
| Daily working minutes | 0.242 | 0.473^***^ | 0.119 |
| Job position | 0.193 | 68.681^**^ | 24.307 |
| Tenure | -0.190 | -24.675^**^ | 8.8105 |
| **Outcome: Sleep quality** | | | |
| Occupational sedentary behaviour | 0.135 | 0.004^*^ | 0.002 |
| Job satisfaction | -0.175 | -0.119^**^ | 0.043 |
| **Outcome: Stress** | | | |
| Occupational sedentary behaviour | 0.076 | 0.001 | 0.000 |
| Job satisfaction | -0.095 | -0.015 | 0.010 |
| Sleep quality | 0.319 | 0.074^***^ | 0.014 |
| Tenure | -0.097 | -0.086 | 0.056 |
| **Outcome: Job satisfaction** | | | |
| Daily working minutes | 0.144 | 0.012^*^ | 0.006 |

**Note.** *β*= Standardised coefficient; *b* = Unstandardised regression coefficient; S.E. = Standard error. Values represent standardised coefficients (β) from the first imputed dataset. Significance testing was based on unstandardised regression coefficients; standardised coefficients are reported for comparison of effect sizes.

**p* ≤ 0.05，***p* ≤ 0.01, ****p* ≤ 0.001

**Table 7a Standardised direct, indirect, and total effects of included variables on stress**

| **Path** | **Direct Effects** | **Indirect Effects** | **Total Effects** |
| --- | --- | --- | --- |
| **Effects of occupational sedentary behaviour on stress via sleep quality** | | | |
| OSB→SLP→STR | 0.076 | 0.043 | 0.119 |
| **Effects of other variables on stress** | | | |
| SLP→STR | 0.319^***^ | - | 0.319^***^ |
| JS→STR | -0.095 | -0.056^*^ | -0.151^*^ |
| TEN→STR | -0.097 | -0.023 | -0.119 |
| JP→STR | - | 0.023 | 0.023 |
| WM→STR | - | 0.006 | 0.007 |

**Note.** OSB=occupational sedentary behaviour, SLP=sleep quality, STR=stress, TEN=tenure, DCI=duration in current industry, JP=job position, WD=workdays per week, WM=daily working minutes, JS=job satisfaction

Values represent standardised coefficients (*β*) from the primary imputed dataset. Significance levels for direct effects were determined based on unstandardised regression coefficients (*b*).

**p* ≤ 0.05，***p* ≤ 0.01, ****p* ≤ 0.001

**Imputed data 3**

Table 6a Unstandardised and standardised path coefficients from the path model

| **Estimator** | ***β*** | ***b*** | ***S.E.*** |
| --- | --- | --- | --- |
| **Outcome: Occupational sedentary behaviour** | | | |
| Daily working minutes | 0.244 | 0.475^***^ | 0.119 |
| Job position | 0.193 | 68.736^**^ | 24.297 |
| Tenure | -0.191 | -24.748^**^ | 8.811 |
| **Outcome: Sleep quality** | | | |
| Occupational sedentary behaviour | 0.135 | 0.004^*^ | 0.002 |
| Job satisfaction | -0.175 | -0.119^**^ | 0.043 |
| **Outcome: Stress** | | | |
| Occupational sedentary behaviour | 0.076 | 0.001 | 0.000 |
| Job satisfaction | -0.095 | -0.015 | 0.010 |
| Sleep quality | 0.319 | 0.074^***^ | 0.014 |
| Tenure | -0.097 | -0.086 | 0.056 |
| **Outcome: Job satisfaction** | | | |
| Daily working minutes | 0.143 | 0.012^*^ | 0.006 |

**Note.** *β*= Standardised coefficient; *b* = Unstandardised regression coefficient; *S.E.* = Standard error. Values represent standardised coefficients (β) from the first imputed dataset. Significance testing was based on unstandardised regression coefficients; standardised coefficients are reported for comparison of effect sizes.

**p* ≤ 0.05，***p* ≤ 0.01, ****p* ≤ 0.001

**Table 7a Standardised direct, indirect, and total effects of included variables on stress**

| **Path** | **Direct Effects** | **Indirect Effects** | **Total Effects** |
| --- | --- | --- | --- |
| **Effects of occupational sedentary behaviour on stress via sleep quality** | | | |
| OSB→SLP→STR | 0.076 | 0.043 | 0.119 |
| **Effects of other variables on stress** | | | |
| SLP→STR | 0.319^***^ | - | 0.319^***^ |
| JS→STR | -0.095 | -0.056^*^ | -0.151^*^ |
| TEN→STR | -0.097 | -0.023 | -0.120 |
| JP→STR | - | 0.023 | 0.023 |
| WM→STR | - | 0.007 | 0.007 |

**Note.** OSB=occupational sedentary behaviour, SLP=sleep quality, STR=stress, TEN=tenure, DCI=duration in current industry, JP=job position, WD=workdays per week, WM=daily working minutes, JS=job satisfaction

Values represent standardised coefficients (*β*) from the primary imputed dataset. Significance levels for direct effects were determined based on unstandardised regression coefficients (*b*).

**p* ≤ 0.05，***p* ≤ 0.01, ****p* ≤ 0.001

**Imputed data 4**

Table 6a Unstandardised and standardised path coefficients from the path model

| **Estimator** | ***β*** | ***b*** | ***S.E.*** |
| --- | --- | --- | --- |
| **Outcome: Occupational sedentary behaviour** | | | |
| Daily working minutes | 0.245 | 0.477^***^ | 0.119 |
| Job position | 0.193 | 68.764^**^ | 24.287 |
| Tenure | -0.191 | -24.821^**^ | 8.807 |
| **Outcome: Sleep quality** | | | |
| Occupational sedentary behaviour | 0.136 | 0.004^*^ | 0.002 |
| Job satisfaction | -0.177 | -0.120^**^ | 0.043 |
| **Outcome: Stress** | | | |
| Occupational sedentary behaviour | 0.075 | 0.001 | 0.000 |
| Job satisfaction | -0.094 | -0.015 | 0.010 |
| Sleep quality | 0.319 | 0.074^***^ | 0.014 |
| Tenure | -0.097 | -0.087 | 0.056 |
| **Outcome: Job satisfaction** | | | |
| Daily working minutes | 0.142 | 0.012^*^ | 0.006 |

**Note.** *β*= Standardised coefficient; *b* = Unstandardised regression coefficient; *S.E.* = Standard error. Values represent standardised coefficients (β) from the first imputed dataset. Significance testing was based on unstandardised regression coefficients; standardised coefficients are reported for comparison of effect sizes.

**p* ≤ 0.05，***p* ≤ 0.01, ****p* ≤ 0.001

**Table 7a Standardised direct, indirect, and total effects of included variables on stress**

| **Path** | **Direct Effects** | **Indirect Effects** | **Total Effects** |
| --- | --- | --- | --- |
| **Effects of occupational sedentary behaviour on stress via sleep quality** | | | |
| OSB→SLP→STR | 0.075 | 0.043 | 0.119 |
| **Effects of other variables on stress** | | | |
| SLP→STR | 0.319^***^ | - | 0.319^***^ |
| JS→STR | -0.094 | -0.056^*^ | -0.151^*^ |
| TEN→STR | -0.097 | -0.023 | -0.120 |
| JP→STR | - | 0.023 | 0.023 |
| WM→STR | - | 0.007 | 0.008 |

**Note.** OSB=occupational sedentary behaviour, SLP=sleep quality, STR=stress, TEN=tenure, DCI=duration in current industry, JP=job position, WD=workdays per week, WM=daily working minutes, JS=job satisfaction

Values represent standardised coefficients (*β*) from the primary imputed dataset. Significance levels for direct effects were determined based on unstandardised regression coefficients (*b*).

**p* ≤ 0.05，***p* ≤ 0.01, ****p* ≤ 0.001

**Imputed data 5**

Table 6a Unstandardised and standardised path coefficients from the path model

| **Estimator** | ***β*** | ***b*** | ***S.E.*** |
| --- | --- | --- | --- |
| **Outcome: Occupational sedentary behaviour** | | | |
| Daily working minutes | 0.244 | 0.476^***^ | 0.119 |
| Job position | 0.193 | 68.753^**^ | 24.292 |
| Tenure | -0.191 | -24.785^**^ | 8.809 |
| **Outcome: Sleep quality** | | | |
| Occupational sedentary behaviour | 0.136 | 0.004^*^ | 0.002 |
| Job satisfaction | -0.177 | -0.120^**^ | 0.043 |
| **Outcome: Stress** | | | |
| Occupational sedentary behaviour | 0.075 | 0.001 | 0.000 |
| Job satisfaction | -0.094 | -0.015 | 0.010 |
| Sleep quality | 0.319 | 0.074^***^ | 0.014 |
| Tenure | -0.097 | -0.087 | 0.056 |
| **Outcome: Job satisfaction** | | | |
| Daily working minutes | 0.143 | 0.012^*^ | 0.006 |

**Note.** *β*= Standardised coefficient; *b* = Unstandardised regression coefficient; *S.E.* = Standard error. Values represent standardised coefficients (β) from the first imputed dataset. Significance testing was based on unstandardised regression coefficients; standardised coefficients are reported for comparison of effect sizes.

**p* ≤ 0.05，***p* ≤ 0.01, ****p* ≤ 0.001

**Table 7a Standardised direct, indirect, and total effects of included variables on stress**

| **Path** | **Direct Effects** | **Indirect Effects** | **Total Effects** |
| --- | --- | --- | --- |
| **Effects of occupational sedentary behaviour on stress via sleep quality** | | | |
| OSB→SLP→STR | 0.075 | 0.043 | 0.119 |
| **Effects of other variables on stress** | | | |
| SLP→STR | 0.319^***^ | - | 0.319^***^ |
| JS→STR | -0.094 | -0.056^*^ | -0.151^*^ |
| TEN→STR | -0.097 | -0.023 | -0.120 |
| JP→STR | - | 0.023 | 0.023 |
| WM→STR | - | 0.007 | 0.007 |

**Note.** OSB=occupational sedentary behaviour, SLP=sleep quality, STR=stress, TEN=tenure, DCI=duration in current industry, JP=job position, WD=workdays per week, WM=daily working minutes, JS=job satisfaction

Values represent standardised coefficients (*β*) from the primary imputed dataset. Significance levels for direct effects were determined based on unstandardised regression coefficients (*b*).

**p* ≤ 0.05，***p* ≤ 0.01, ****p* ≤ 0.001
